# Predominant Role of Host Genetics in Controlling the Composition of Gut Microbiota

**DOI:** 10.1371/journal.pone.0003064

**Published:** 2008-08-26

**Authors:** Zaruhi A. Khachatryan, Zhanna A. Ktsoyan, Gayane P. Manukyan, Denise Kelly, Karine A. Ghazaryan, Rustam I. Aminov

**Affiliations:** 1 Laboratory of Molecular Genetics, Institute of Molecular Biology of Armenian National Academy of Sciences, Yerevan, Armenia; 2 Group of Molecular and Cellular Immunology, Institute of Molecular Biology of Armenian National Academy of Sciences, Yerevan, Armenia; 3 Rowett Institute of Nutrition and Health, University of Aberdeen, Aberdeen, United Kingdom; University of Queensland, Australia

## Abstract

**Background:**

The human gastrointestinal tract is inhabited by a very diverse symbiotic microbiota, the composition of which depends on host genetics and the environment. Several studies suggested that the host genetics may influence the composition of gut microbiota but no genes involved in host control were proposed. We investigated the effects of the wild type and mutated alleles of the gene, which encodes the protein called pyrin, one of the regulators of innate immunity, on the composition of gut commensal bacteria. Mutations in *MEFV* lead to the autoinflammatory disorder, familial Mediterranean fever (FMF, MIM249100), which is characterized by recurrent self-resolving attacks of fever and polyserositis, with no clinical signs of disease in remission.

**Methodology/Principal Findings:**

A total of 19 FMF patients and eight healthy individuals were genotyped for mutations in the *MEFV* gene and gut bacterial diversity was assessed by sequencing 16S rRNA gene libraries and FISH analysis. These analyses demonstrated significant changes in bacterial community structure in FMF characterized by depletion of total numbers of bacteria, loss of diversity, and major shifts in bacterial populations within the *Bacteroidetes, Firmicutes* and *Proteobacteria* phyla in attack. In remission with no clinical signs of disease, bacterial diversity values were comparable with control but still, the bacterial composition was substantially deviant from the norm. Discriminant function analyses of gut bacterial diversity revealed highly specific, well-separated and distinct grouping, which depended on the allele carrier status of the host.

**Conclusions/Significance:**

This is the first report that clearly establishes the link between the host genotype and the corresponding shifts in the gut microbiota (the latter confirmed by two independent techniques). It suggests that the host genetics is a key factor in host-microbe interaction determining a specific profile of commensal microbiota in the human gut.

## Introduction

The human gut microbiota has been shaped by the long co-evolutionary history of symbiotic host-microbe interaction [1] and has evolved to play an important role in maintaining human health by preventing colonization by pathogens, degrading dietary and *in situ*-produced compounds, producing nutrients, and shaping and maintaining the normal mucosal immunity [2]–[4]. Recently, other important functions of commensal microbiota became apparent including an anti-inflammatory effect on gut epithelium [5], [6], the influence on lipid metabolism of the host [7] and association with obesity [8], [9] as well as the involvement in intestinal homeostasis, repair and angiogenesis [10], [11].

Recently, the intricate molecular mechanisms behind this host-microbe cross-talk began to emerge. The innate immune system of the mammalian host senses bacteria through an impressive array of receptors, called Toll-like (TLR) and Nod-like (NLR) (also called CATERPILLER, NOD-LRR, and NACHT-LRR) receptors. The first mammalian TLR was discovered a decade ago [12] and the functionality of the first NLRs (then called NODs) as sensors of bacterial ligands was established in 2001 [13]. TLRs are predominantly but not exclusively located in the transmembrane region of host cells, while NLR proteins are mostly located in the cytoplasm [14]. Both groups of receptors contain leucine-rich repeats (LRR), which are involved in detection of danger signals and, in particular, of whole bacteria and bacterial products through the recognition of pathogen associated molecular patterns (PAMP). PAMP signalling through the innate immunity receptors appeared to be important not only for the recognition of, and launching defences against, the invading pathogens but also for the maintenance of intestinal epithelial homeostasis and for protection against gut injury and associated mortality [11]. Bacterial products are also crucial for the normal immune system development; for example, a single bacterial polysaccharide (PSA) from a commensal bacterium *Bacteroides fragilis* was shown is capable of directing the complete cellular and physical maturation of the developing immune system [15]. In murine experimental colitis models, signalling by probiotic bacterial DNA ligands through TLR9 exerted a strong anti-inflammatory effect [16] while signalling through TLR2 seems important for regulation of mucosal inflammation and maintenance of intestinal epithelial barrier integrity [17], [18].

Another type of regulation of the host metabolism by microbes can be through the low molecular weight bacterial metabolites such as short chain fatty acids (SCFAs) and, in particular, butyrate. Butyrate is the preferred energy source for the colonic epithelium and may play an important role in colonic health and prevention of colorectal cancer and colitis [19]–[21].

Because of limitations imposed by the extreme diversity of microbiota in the conventional gut, which does not allow monitoring the behaviour of a single bacterium, much less is known of how the bacterial counterpart responds to the host-generated molecular signals. Initially, the bacterial response was considered to be limited to various strategies used by gut bacteria to forage the endogenously produced substrates of the host [22], [23]. Interestingly, however, one of the aspects of this interaction, namely the specific fucosylation, seems important for both sides involved in the host-bacterial dialogue. On one side, the intestinal bacteria induce mucin fucosylation in the gut epithelium thus creating a specific nutritional econiche [22], [24]. On the other side, fucosylation of the cell surface in a mammalian-like fashion appeared to be essential for the gut bacteria to establish the successful mutualistic relationship with the host. The lack of this property may result in the failure of bacteria to colonize the mammalian intestine [25]. Yet another “language” of host-bacterial communication could be through the quorum sensing (QS), which utilizes hormone-like compounds and, in *E. coli*, has been implicated in a potential cross-communication between the luxS/AI-3 bacterial QS system and the epinephrine host signalling system [26]. Bacteria may also sense the immune status of the host and respond by the expression of a quorum-sensing dependent virulence determinant [27]. These two examples of quorum-sensing-mediated signalling between the host and bacteria are taken from the pathogenic bacteria but it cannot be excluded that this type of communication is common among gut symbiotic bacteria as well.

What happens if this fine-tuned molecular cross-talk is compromised and the host fails to recognize bacterial components as, for example, in the case of inflammatory bowel disease (IBD) such as Crohn's disease (CD)? In a subset of CD patients, mutations in the NOD2/CARD15 protein have been associated with susceptibility to the disease [28], [29]. Bacterial ligand for this receptor has been recently identified as bacterial muramyl dipeptide [30], [31] and failure to recognise this ligand in CD patients leads to the breach of tolerance and to the launch of an aggressive Th1-skewed response against the antigens of normal diet and commensal bacteria as well as to production of autoantibodies [32]–[34]. The microbiota of these patients demonstrates diminished diversity, in particular, due to the depletion of bacteria belonging to the *Firmicutes* and *Bacteroidetes* phyla [35], [36]. Interestingly, the loss of the transcription factor T-bet, which regulates the innate immune system, influences bacterial populations in the murine gut in a way that they become colitogenic and this colitis is communicable to genetically intact hosts [37]. Presently, it is not clear how the composition of the normal microbiota is restructured to become a “pathobiota” but, nevertheless, this is the first experimental proof of the view that the community as a whole may demonstrate pathogenic properties, not a single pathogenic bacterium.

In our study, we investigated the effect of mutations in a single gene of the host on commensal gut microbiota. The gene under investigation, *MEFV* (for MEditerranean FeVer), encodes a protein called pyrin/marenostrin, which is involved in regulation of innate immunity [38]. Structurally, this 781-residue protein consists of a 92-amino acid N-terminal PYRIN (PYD) domain, a B-box zinc finger, a coiled-coil region and a ∼200-amino acid C-terminal B30.2/rfp/SPRY domain [39]. Mutations in this gene lead to an autoinflammatory disorder, familial Mediterranean fever (FMF, MIM249100), which is characterised by short recurrent self-resolving attacks of fever and polyserositis, with no clinical signs of disease in remission periods. The carrier rate and occurrence of disease is high in populations originating from the Mediterranean basin, including Sephardic Jews, Armenians, Arabs, and Turks. Articles describing positional cloning of the gene by two independent consortia appeared in 1997 [40], [41] and to date 166 *MEFV* mutations and single nucleotide polymorphisms (SNPs) are detected in the gene (http://fmf.igh.cnrs.fr/infevers). The hotspot of *MEFV* mutations is localized in exon 10, which encodes the B30.2/rfp/SPRY domain, and this single locus inheritance greatly facilitated our analysis of host genetics in comparison with other disorders with more complex genetics. Genome-wide scans for IBD susceptibility, for example, have resulted in identification of seven loci that are confirmed and replicated in several studies, but with many more that need independent confirmation and verification [42].

The present work is the first attempt to analyze simultaneously the genetics of both sides involved in the host-gut microbiota relationship. All study participants were analyzed for *MEFV* mutations and the composition of gut microbiomes was assessed by two culture-independent techniques, fluorescent in situ hybridization (FISH) and 16S rDNA gene library analyses. For the first time, our findings conclusively establish the key role of the host genotype in host-microbe interaction, which determines a highly specific composition of commensal microbiota in the human gut.

## Results

### MEFV Genotyping

Nineteen FMF patients with clinically confirmed disease and eight healthy individuals were subjected to genotyping (Table 1). We performed genotyping of the exon regions of the *MEFV* gene, which is thought to be a single genetic determinant of FMF, in particular exons 2 and 10, where the majority of disease-associated mutations were identified (http://fmf.igh.cnrs.fr/infevers). FMF-associated mutations were found in all FMF patients displaying the clinical signs of the disease, as well as in one healthy Armenian control subject (in a simple heterozygous state) without disease-related symptoms (Table 1). All mutations were located in exon 10, only synonymous substitutions were detected in exon 2, irrespectively of the health status. The majority of mutations in FMF patients were M694V, which were present in the compounded form with other disease alleles in eight patients and in a simple heterozygous form in six patients. In the latter group, no other disease-related mutations in exon 2, except neutral SNPs, were detected. Homozygotes were represented exclusively by the M680I mutation (Table 1). Interestingly, the SNPs in the control group were all in the heterozygous state, while the homozygous forms were encountered only among FMF patients.

**Table 1:** *MEFV* genotypes of FMF patients and control subjects and gut microbiota analysis methods applied.

Subjects	Exon 10 FMF mutations	Exon 2 non FMF mutations		Gut microbiota analyses
		D102D	G138G	
C1b	N/D*	HT	HT	S
C4b	N/D	N/D	N/D	S; F
C6b	M680I	HT	HT	S; F
C11b	N/D	N/D	N/D	S; F
C15b	N/D	HT	HT	S; F
C16b	N/D	N/D	N/D	F
C18b	N/D	HT	HT	S; F
Cm13b	N/D	N/D	N/D	S; F
FMF 0	M680I/V726A	N/A**	N/A	SR
FMF 1	M694V/A761H	N/A	N/A	SR
FMF 2	M694V/A761H	N/A	N/A	SR
FMF 3	M694V/A761H	N/A	N/A	SR
FMF 5	M680I/M694V	N/A	N/A	SR
FMF 7	M694V	N/D	N/D	SR
FMF 8	M694V	HM	HM	SR
FMF 10	M694V	HT	HT	SR; FR
FMF 11	M694V	HT	HT	SR
FMF 12	M694V	HM	HM	SR; FR
FMF 13-123	M680I/V726A	N/A	N/A	SA; SR; FA; FR
FMF 14	M694V/A761H	N/A	N/A	SR; FR
FMF 87	M694V/A761H	HT	HT	FR; FA
FMF 103	M680I/M680I	N/A	N/A	SA; FA
FMF 109	M694V	HT	HT	FA
FMF 110	M694V/V726A	N/D	N/D	FA
FMF 111	M680I/M694V	HM	HM	FA
FMF 114	M680I/M680I	N/A	N/A	FR
FMF 124	M680I/M680I	N/A	N/A	SA; FA

Control subjects denoted with prefix C; FMF patients denoted with prefix FMF.

Abbreviations: S, 16S library; F, FISH analysis; SR, 16S library in FMF remission; SA, 16S library in FMF attack; FR, FISH analysis in FMF remission; FA, FISH analysis in FMF attack; HT, heterozygote; HM, homozygote.

* N/D, not detected.

** N/A, not analyzed.

### Phylogenetic and comparative analysis of 16S libraries

Three 16S rDNA libraries from the fecal samples of healthy controls as well as of FMF patients in remission and disease attack were generated (designated as S, SR and SA in Table 1). After the quality control a total of 1328 validated sequences (572 for healthy controls, 629 for FMF remission and 127 for FMF attack) were analyzed and phylogenetic analysis was performed to establish taxonomic positioning of sequences obtained (Table 2). Among 1328 clones analyzed, there were 268 distinct OTUs and the vast majority (95.95%) fell into the two major phyla, *Bacteroidetes* with 99 OTUs (38.15%) and 714 sequences (53.76%) and *Firmicutes* with 158 OTUs (57.8%) and 580 sequences (43.67%). *Proteobacteria* were represented by 9 OTUs (3.47%) and 30 sequences (2.26%). Only four *Actinobacteria* sequences (2 OTUs) were detected, which were all derived from one FMF patient in remission.

**Table 2:** Phylogenetic distribution of 16S rRNA gene sequences and phylotypes retrieved from healthy individuals and FMF patients in remission and attack.

Phylum	Healthy		FMF remission		FMF attack	
	OTUs*	Clones	OTUs	Clones	OTUs	Clones
*Bacteroidetes*	54	324	57	319	21	71
% *Bacteroidetes*	40.91	56.64	35.19	50.72	40.38	55.91
*Firmicutes*	73	237	98	289	29	54
% *Firmicutes*	55.30	41.43	60.49	45.95	55.77	42.52
*Proteobacteria*	5	11	5	17	2	2
% *Proteobacteria*	3.79	1.92	3.09	2.70	3.85	1.57
*Actinobacteria*	0	0	2	4	0	0
% *Actinobacteria*	0	0	1.23	0.63	0	0
Total	132	572	162	629	52	127

* OTUs were defined at 99% similarity level.

In all three libraries, bacteria belonging to the *Bacteroidetes* phylum were the most abundant, containing more than the half of the sequences in each library (from 50.72% to 56.64%) but a relatively smaller proportion of OTUs (from 35.19% to 40.91%), whereas the *Firmicutes* phylum had the larger proportion of phylotypes (from 55.30% to 60.49%) with the less copious sequence numbers (from 41.43% to 45.95%) (Table 2). At the phylum level, the relative proportions of *Bacteroidetes* and *Firmicutes* were not significantly different among the three libraries, however, pairwise comparisons of each 16S rDNA library to every other library by using the online library compare tool at RDP-II (Naive Bayesian rRNA Classifier) revealed significant differences within the lower taxa levels (Table 3). In particular, in asymptomatic FMF patients in remission, the proportion of *Enterobacteriaceae*, *Acidaminococcaceae*, *Ruminococcus* and *Megasphaera* was significantly increased in comparison with control subjects, while *Roseburia* was significantly reduced (Table 3). In the active disease, the proportion of *Prevotellaceae, Dialister* and *Prevotella* was significantly lower than in healthy controls while the *Porphyromonadaceae*, *Phascolarctobacterium*, *Faecalibacterium*, and *Parabacteroides* were significantly increased (Table 3). Interestingly, there were also differences due to the disease state (remission or attack) and the affected bacterial groups were *Acidaminococcaceae*, *Porphyromonadaceae*, *Megasphaera*, *Dialister*, *Faecalibacterium*, and *Parabacteroides* (Table 3).

**Table 3:** Comparison of 16S rRNA gene libraries derived from healthy controls and FMF patients in remission and attack.

Bacterial taxa	Control	Remission	Attack
Class *Gammaproteobacteria* *	0.2%	2.1%	0%
Order *Enterobacteriales* *	0%	1.6%	0%
Family *Enterobacteriaceae* *	0%	1.6%	0%
Family *Acidaminococcaceae* * ^,^ ***	9.2%	14.8%	3.1%
Family *Prevotellaceae* **	28.8%	24.5%	17.3%
Family *Porphyromonadaceae* ** ^,^ ***	2.3%	3.3%	9.4%
Genus *Ruminococcus* *	0.2%	2.8%	1.6%
Genus *Roseburia* *	2.8%	0.8%	2.4%
Genus *Megasphaera* * ^,^ ***	0.2%	4.5%	0%
Genus *Dialister* ** ^,^ ***	7.9%	6.7%	0%
Genus *Phascolarctobacterium* **	0%	0.6%	3.1%
Genus *Faecalibacterium* ** ^,^ ***	6.5%	6.5%	14.2%
Genus *Prevotella* **	28.4%	23.1%	16.5%
Genus *Parabacteroides* ** ^,^ ***	1.2%	2.1%	5.9%

* p<0.01, comparison of remission vs. control libraries.

** p<0.01, comparison of attack vs. control libraries.

*** p<0.01, comparison of remission vs. attack libraries.

### Community analysis of 16S rDNA libraries

The estimates of diversity, richness, evenness and library coverage for the three 16S rDNA clone libraries studied are shown in Table 4. At the phylotype level, the most diverse gut bacterial community was observed in FMF patients in remission. The slightly lower indices were found for the healthy microbiota, while the bacterial diversity during disease attacks was consistently lower. The similar trend was observed for another community parameter, species richness, which was the highest in the gut of FMF patients in remission and in healthy volunteers but much lower during disease attacks. On the contrary, evenness index, which describes how evenly the individual sequences are represented in phylotypes, was the highest in the disease attack community (Table 4). Good's coverage was 89.35–90.73% for the sequence sets from FMF patients in remission and healthy controls but only 77.2% for sequences from FMF patients in attack reflecting the lower number of subjects and sequences collected.

**Table 4:** Indices of diversity, richness and evenness and library coverage for 16S rRNA gene libraries.

Measurement	Healthy (n = 7)	FMF remission (n = 12)	FMF attack (n = 3)
No. of clones	572	629	127
No. of OTUs	132	162	52
Singletons	53	67	29
Doubletons	18	33	9
Chao1 estimator of species richness*	204.5 (168, 280)	227 (197, 283)	92.5 (68, 156)
Shannon diversity index (H)	4.3	4.5	3.5
Simpson diversity index (1-D)	0.978	0.984	0.96
Simpson reciprocal index (1/D)	45.45	62.5	25
Evenness index	0.558	0.556	0.637
Good's estimator of coverage, %	90.73	89.35	77.2

Calculations were made based on OTU definition at 99% sequence identity.

* The values in parentheses are the 95% confidence intervals, which assess the precision of the richness estimates.

**Figure 1:**
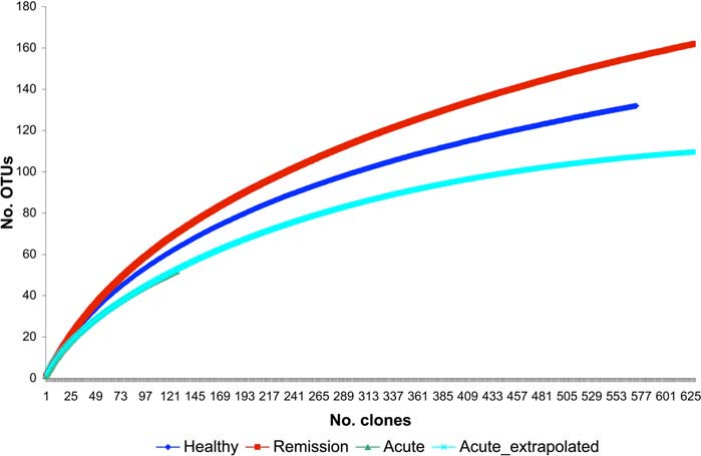
Rarefaction curves obtained for clone libraries from healthy controls and FMF patients. The rarefaction curves were generated by plotting the number of phylotypes (defined at 99% level) against the number of clones sequenced. The shape of the curves of observed phylotype richness indicates a trend of diminishing chance of finding new phylotypes as the sampling continues. Data for library in attack were extrapolated by empiric regression function a×x^2^b×exp(−c×x). Coefficient of regression determination (R^2^) was 99.9%. Regression statistics: adjusted R^2^ – 0.9991; standard error – 0.1720; a = 1.8690; b = 0.7104; c = 8.0466E-04. Healthy controls are shown in dark blue; FMF patients in remission – in red and attack – in green, the extrapolated curve is shown in light blue.

Rarefaction analysis of clone libraries essentially confirmed these findings and suggested that FMF patients in remission possess the most diverse gut bacterial community, which is even higher than in healthy subjects (Fig. 1). Because of limited number of sequences in the clone library from the acute disease, we extrapolated the curve using an empiric regression function (for details, see the footnote to Fig. 1). The extrapolated curve confirmed the initial findings suggesting diminished diversity of gut bacteria in FMF attack in comparison with disease remission and control.

**Figure 2:**
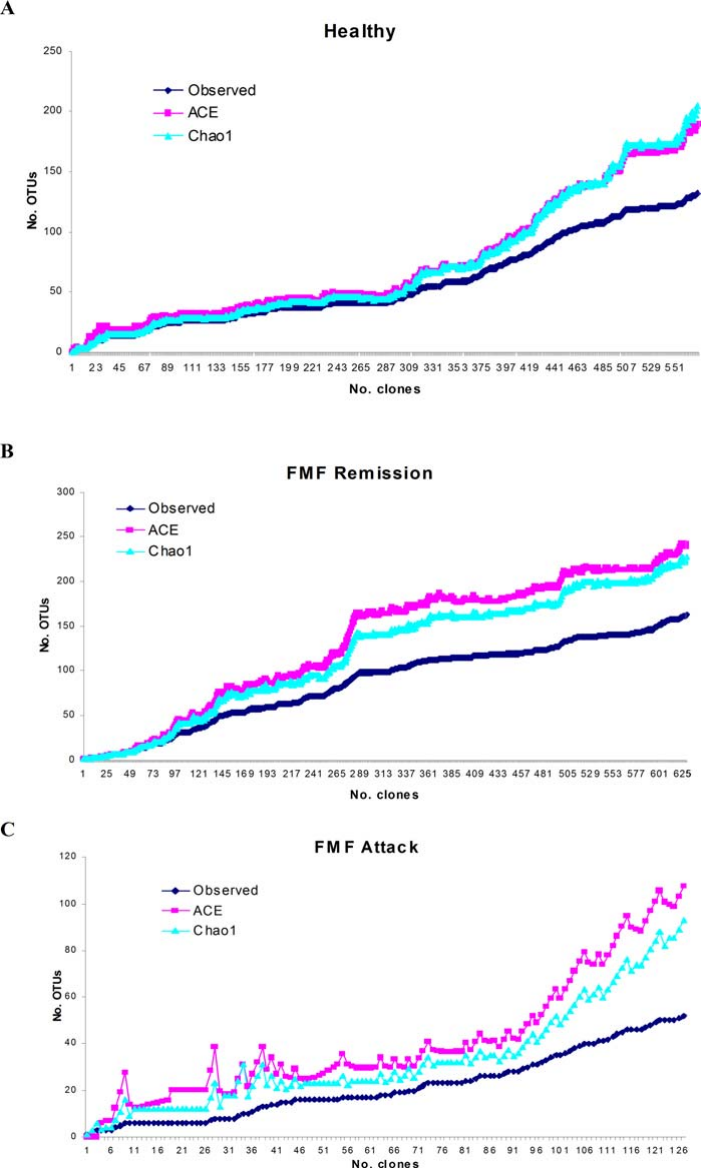
Collector's curves of the observed and estimated phylotype richness of 16S libraries. Collector's curves of the observed (shown in dark blue) and estimated (Chao1 (light blue) and ACE (red)) phylotype richness calculated for healthy subjects (A), FMF remission (B), and FMF attack (C) with the phylogenetic depth defined at 99% level.

Collector's curves of the observed and estimated phylotype richness are shown in Fig. 2. In healthy subjects, both estimates, Chao1 and abundance-based coverage estimator (ACE), were highly similar and, up to the point of collection of ca. 320 clones, were in a steady and congruent increase together with the observed phylotype number (Fig. 2A). In this sampling area, almost any new clone sequence added represented a novel phylotype. Adding new sequences after this point, however, led to the gap expansion between the estimated and observed phylotype numbers suggesting repeated sampling of the phylotypes already present in the sample. In FMF remission, the gap between the observed and estimated phylotype richness was constant after the sampling point of 290 clones (Fig. 2B). Confirmatory to the previous analyses, the phylotype number estimates at the sampling point with 500 clones gave the higher numbers of phylotypes in FMF remission than in the norm (220 vs. 160). The difference between the estimates and observed phylotype richness is highest in acute FMF possibly reflecting the insufficient coverage in this group (77.2%).

### FISH analysis

Twelve Cy3-labelled oligonucleotide probes that target the total and specific predominant groups of human gut bacteria were used in this study to enumerate bacteria in the feces of FMF patients and healthy controls (for details, see Materials and Methods). Hybridization signals were detected for all bacterial groups, indicating their presence as dominant microbiota in all fecal samples analyzed.

FISH analysis revealed that the composition of fecal bacteria in FMF patients in both inactive and active phases of the disease is different from that of the healthy controls. The results of enumeration of bacterial cells in each group and their relative proportions versus the universal bacterial probe Eub338 are summarized in Table 5. All fecal samples were processed exactly in the same way and the count of cells hybridized with Eub338 can be calculated to give the absolute numbers of bacteria per gram of feces. No significant difference in concentration of total fecal bacteria was found between the healthy controls and FMF patients in remission, the mean values 5.47×10^10^/g and 5.12×10^10^/g, respectively (Table 5). In the acute stage of the disease, however, the concentration of bacteria in fecal material was substantially lower, at 3.56×10^10^/g.

**Table 5:** Mean numbers of predominant bacteria in fecal samples of FMF patients in both stages of the disease and healthy controls detected by FISH.

Probes	Controls (n = 7)		FMF patients			
			Remission (n = 6)		Attack (n = 7)	
	Bacterial number/g*	% bacteria**	Bacterial number/g	% bacteria	Bacterial number/g	% bacteria
Eub338	547.2±315.5	100	512.4±250.4	100	356.1±83.5	100
Bac303	151.3±106.0	27.3±9.5	185.9±136.0	39.1±18.7	135.4±55.6	38.6±15.7
Erec482	108.5±60.6	21.1±5.3	101.0±69.8	18.0±8.0	100.8±37.0	28.9±9.6
Rrec584	45.1±54.2	6.5±4.1	44.3±35.5	8.3±4.5	60.3±53.0	18.0±14.1
Fprau645	54.4±19.6	11.3±3.2	49.8±38.3	10.4±6.1	53.2±47.2	13.8±8.6
Rfla729/Rbro730	59.0±45.0	9.9±3.2	28.5±35.7	5.3±4.7†	8.5±9.3††	2.6±2.8††
Prop853	64.2±51.7	10.8±3.2	44.3±56.2	7.1±7.6	12.8±10.4††	3.5±2.5††
Bif164	20.0±27.3	2.7±3.1	27.0±44.6	4.6±6.1	5.0±5.9	1.7±2.3
Ato291	42.2±24.9	7.8±4.1	28.9±20.6	6.3±3.0	16.9±12.7†	4.5±2.9
EnterobactD	5.4±3.0	1.2±0.6	4.6±3.6	1.2±1.5	7.9±12.0	1.9±2.9
Lab158	2.0±3.1	0.4±0.4	1.9±2.4	0.3±0.3	1.3±2.0	0.4±0.8

Mean count±SD.

* Counts 10^8^ cells per gram of fecal specimen.

** The percentage relative to the total number of cells determined by Eub338.

† p<0.05 as compared to healthy controls.

†† p<0.01 as compared to healthy controls.

Among the taxonomic groups tested, the highest proportion was detected with the Bac303 probe, which targets the *Bacteroides* group. It gave the concentration of these bacteria at 1.51×10^10^/g (27.3% of total bacteria) in healthy controls, with elevation in FMF patients to 1.86×10^10^/g (39.1%) in remission and to 1.35×10^10^/g (38.6%) in the attack period (Table 5). Cluster XIVa bacteria (*Clostridium coccoides* group) was the second most abundant group enumerated with the Erec482 probe comprising 1.09×10^10^/g (21.1%) in the healthy controls and approximately 1.01×10^10^/g in both, FMF remission and attack phases (18% and 28.9% of total bacteria, respectively). The *E. rectale-Roseburia* probe Rrec584, which is a nested probe within the *C. coccoides* group (enumerated with Erec482), detected 6.5%, 8.3% and 18.0% within the total bacteria probe, Eub338, and 41.5%, 43.8% and 59.8% within the Erec482 probe in healthy controls, FMF remission and FMF attacks, respectively. There were significant populations of *Fecalibacterium prausnitzii* related bacteria (Fprau645), the second important group of butyrate producers within clostridial cluster IV, in all studied groups, with the tendency to increase in acute FMF. These bacteria comprised 0.54×10^10^/g (11.3%) in healthy controls, 0.5×10^10^/g (10.4%) in FMF remissions, and 0.53×10^10^/g (13.8%) in acute stage of FMF. Another group within clostridial cluster IV, ruminococci, which was detected with the combination of probes Rfla729 and Rbro730, demonstrated lower levels in FMF patients, especially in the acute phase of the disease, compared to healthy controls. In particular, the members of the *R. flavefaciens*/*R. bromii* group accounted for 0.59×10^10^/g (9.9%) in healthy controls, 0.29×10^10^/g (5.3%) in FMF remissions, and 0.09×10^10^/g (2.6%) in acute FMF. Similarly, clostridial cluster IX representatives, detected with the probe Prop853, were estimated to be at 0.64×10^10^/g (11%) in healthy group, whereas in FMF remission these bacteria were reduced to 0.44×10^10^/g (7.1%), with even more significant reduction in the acute stage of FMF, accounting for 0.13×10^10^/g (3.5%). *Atopobium* group also tended to be less represented in gut microflora of FMF patients compared with healthy subjects, steadily decreasing from 0.42×10^10^/g (7.8%) in healthy controls to 0.29×10^10^/g (6.3%) in remission and 0.17×10^10^/g (4.5%) in attack. Members of the *Bifidobacterium*, *Enterobacteriaceae* and *Lactobacillus-Enterococcus* groups, which were detected by the probes Bif164, EnterobacD and Lab158, respectively, did not reveal significant differences between the studied groups because they were found in low numbers and demonstrated high inter-individual variability (Table 5).

We also attempted to evaluate the difference in gut microbiota composition in remission and attack periods of disease in the “pure” form, when the genetic, phenotypic and environmental variables are removed from the analysis. For this, we monitored the intra-individual shifts in total and main gut bacterial populations during the remission and attack phases of the disease in two patients and these observations confirmed and reinforced the tendencies found between the larger combined cohorts of FMF sufferers in the remission and acute stages (Table 6). First, the total numbers of bacteria in the gut enumerated with the Eub338 probe decreased about two-fold. Second, in a number of bacterial groups, the tendencies detected earlier became more profound and statistically significant. In particular, these changes included a dramatic increase in concentration of cluster XIVa bacteria and particularly of *E. rectale*-*Roseburia* cluster during the acute phase of the disease, with the reduction in concentration of cluster IX bacteria and ruminococci. The fall of proportion of populations of lactic acid bacteria enumerated with the Bif164 and Lab158 probes during the disease attack was significant in one patient, FMF 13-123, while in the other, FMF 87, the differences in this group were not significant. Thus, the monitoring of intra-individual bacterial shifts during the remission and attack phases validated and confirmed the findings involving the larger cohorts (Tables 5 and 6).

**Table 6:** Distribution of main bacterial groups in two FMF patients in remission and attack.

Probes	Patient FMF 87		P-value	Patient FMF 13-123		P-value
	Remission	Attack		Remission	Attack	
Eub338*	100% (684.08±94.01)	100% (318.17±69.16)	0.0001	100% (656.03±144.49)	100% (389.53±86.23)	0.0001
Bac303	65.21±14.05	58.23±11.55	0.0037	26.02±7.11	25.01±6.17	0.2858
Erec482	12.32±3.5	26.63±5.9	0.0001	27.91±5.21	45.12±14.64	0.0001
Rrec584	4.29±1.02	18.04±4.08	0.0001	13.63±3.03	43.91±9.28	0.0001
Fprau645	8.51±2.53	12.0±3.24	0.0001	18.46±3.67	16.80±3.69	0.0092
Rfla729/Rbro730	0.57±0.25	0.31±0.13	0.0012	3.90±1.32	0.75±0.37	0.0001
Prop853	2.64±0.60	1.97±0.55	0.0010	5.22±1.16	0.37±0.22	0.0001
Bif164	1.82±0.42	0.59±0.22	0.0001	3.31±0.73	0.16±0.11	0.0001
Ato291	2.30±0.65	3.91±1.23	0.0001	4.06±0.86	3.59±1.33	0.0262
EnterobactD	0.50±0.1	0.32±0.12	0.0006	0.65±0.18	1.01±0.72	0.0013
Lab158	0.81±0.23	0.67±0.24	0.1617	0.64±0.16	N/D†	–

Mean count of the percentage relative to the total number of cells determined by Eub338 ± SD.

* The values in parentheses are the absolute numbers of cells determined by Eub338 ± SD (counts 10^8^ cells per gram of fecal specimen).

† N/D, not detected.

The part of FMF patients was undergoing colchicine therapy and we compared the composition of gut bacteria in respect to this treatment (Table 7). There was no difference in acute disease but in remission a certain tendency to normalization was observed in the *Bacteroides* group and in clostridial cluster IX bacteria. Nevertheless, these differences were not statistically significant and the data sets of colchicine-treated and untreated patients were combined together for the microbiota analyses.

**Figure 3:**
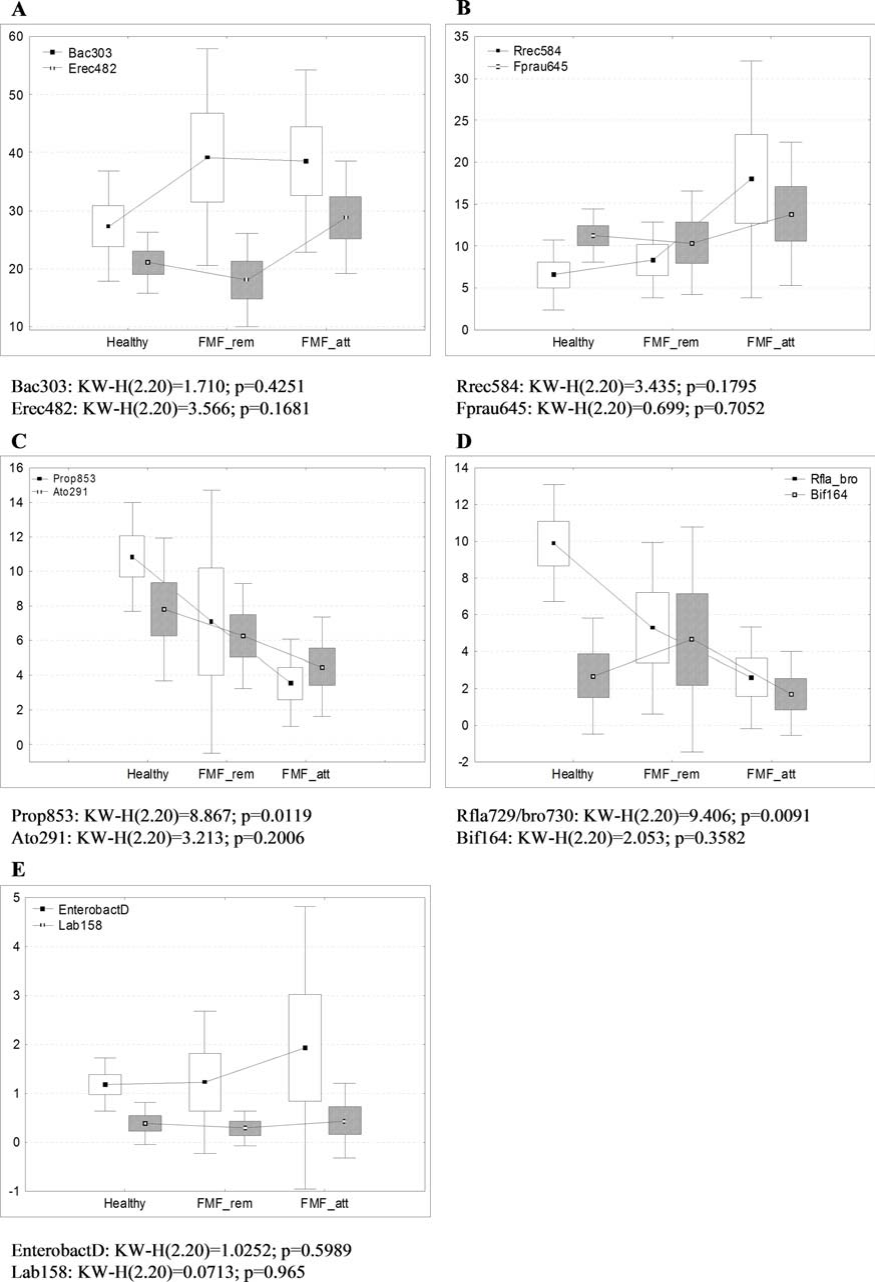
Comparison of the main bacterial groups identified by FISH between healthy and diseased groups. Comparative analysis of relative proportions of bacterial groups detected by different oligonucleotide probes in fecal samples between FMF patients in remission and attack periods and healthy control group (by Kruskal-Wallis test for multiple comparisons). On box-and-whisker plots the points within boxes indicate the mean values of bacterial proportions, the boxes represent standard errors (SE) and the vertical bars represent standard deviations (SD).

Multiple comparative analyses of relative proportions of bacterial populations in the gut of the three cohorts were performed using the Kruskal-Wallis test and the results are presented in Fig. 3. The proportion of *Bacteroides* was significantly higher in both diseased cohorts in comparison with the healthy controls, while the proportion of cluster XIVa bacteria was only increased in the acute phase of the disease in comparison with the control group and remission (Fig. 3A). The fraction of *E. rectale-Roseburia* cluster among total bacteria was substantially increased, especially in active disease (Fig. 3B), while bacteria of cluster IX and *Atopobium* decreased in the attack phase of the disease in comparison with controls and remission (Fig. 3C). The proportion of ruminococci was significantly lower in FMF patients (Fig. 3D), while no significant differences were detected among *F. prausnitzii*, bifidobacteria, lactic acid bacteria, and enterobacteria (Fig. 3B, 3D and 3E).

**Table 7:** Distribution of main bacterial groups in respect to colchicine treatment.

Probes	FMF remission				FMF attack			
	Colchicine treatment (n = 3)		No colchicine treatment (n = 3)		Colchicine treatment (n = 3)		No colchicine treatment (n = 4)	
	Bacterial number/g*	% Bacteria**	Bacterial number/g	% bacteria	Bacterial number/g	% bacteria	Bacterial number/g	% bacteria
Eub338	668.7±21.6	100	356.0±288.0	100	385.9±22.8	100	333.7±109.7	100
Bac303	166.1±41.1	25±6.8	205.7±208.2	53.3±15.0	146.5±68.9	38.7±20.5	127.1±53.0	38.5±14.5
Erec482	156.0±42.0	23.5±6.9	46.0±36.5	12.6±4.7	110.9±56.6	28.8±14.5	93.2±20.8	28.9±6.8
Rrec584	69.0±34.6	10.4±5.4	19.6±11.3	6.2±2.7	78.1±83.0	20.4±21.2	46.9±21.6	16.2±9.5
Fprau645	62.3±51.2	9.4±7.9	37.3±24.0	11.3±5.4	42.5±20.1	11.0±5.2	61.2±63.1	15.8±10.8
Rfla729/Rbro730	50.4±41.8	7.4±5.9	6.6±2.3	3.2±2.5	11.0±12.7	2.8±3.1	6.6±7.3	2.4±3.0
Prop853	79.3±64.5	11.7±9.1	9.3±7.8	2.5±0.2	14±15.8	3.5±3.8	11.9±7.1	3.5±1.7
Bif164	46.4±61.8	6.8±8.9	7.5±4.4	2.5±1.2	3.4±5.4	0.9±1.5	6.2±6.8	2.3±2.8
Ato291	41.8±23.4	6.2±3.2	16.0±4.4	6.3±3.5	24.7±16.2	6.3±3.8	11.0±6.5	3.1±1.1
EnterobactD	4.5±3.2	0.7±0.5	4.8±4.7	1.8±2.0	13.2±17.3	3.3±4.2	4.0±6.3	0.9±1.2
Lab158	1.9±1.9	0.3±0.3	1.8±3.2	0.3±0.5	0.2±0.2	0.1±0.1	2.0±2.5	0.7±1.0

Mean count±SD.

* Counts 10^8^ cells per gram of fecal specimen.

** The percentage relative to the total number of cells determined by Eub338.

**Figure 4:**
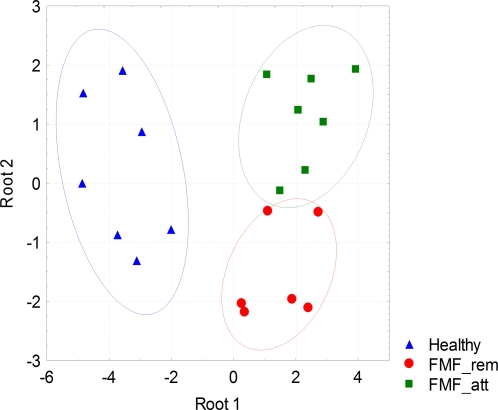
Discriminant function analysis of FISH data. Number of variables in model: 10; grouping - 3 groups. Wilks's lambda coefficient – 0.045; F-test - (20,16) = 2.95, p<0.016. Root 1, 2 – discriminant functions 1 and 2 (1^st^ and 2^nd^ canonical roots). Regions of group distribution are outlined by ellipses; healthy is shown in blue triangles, remission – in red circles, and attack – in green squares.

Discriminant function analysis (DA) is a powerful tool to extract specific patterns in multiple datasets and we used this approach to analyze the specific gut microbiota distribution patterns in our three cohorts. The summary of discriminant function analysis with 20 subjects and 10 variables is shown in Fig. 4. It revealed three clusters in bacterial distribution that are highly specific, well separated and distinct for healthy controls, FMF remission, and FMF attack. As shown in Table 8, the total percentage of correct classification was 95%, thus 95% of total subjects were classified into the correct groups (100% in healthy controls, 83.3% in remission and 100% in attack). The validity of the variables in differentiating the groups was supported by the Wilks's lambda coefficient, which may vary from 0 to 1 (the smaller the lambda, the more the variable differentiates the groups), and in this case it had a value of 0.045. The F-test of Wilks's lambda, which is used to test if the discriminant model as a whole is significant, produced (20,16) = 2.95; p<0.016. There was only one case of misclassification from a total of 20 when the remission stage was assessed as acute. This patient refused the colchicine therapy and was suffering from very frequent FMF attacks and there is a possibility that at the time of sampling the microbiota was not restructured back to the composition specific for the remission stage. Thus the model displayed good predictive accuracy and suggested the specific patterns of gut microbiota distribution in the healthy and diseased states.

**Table 8:** Classification table of correct estimates based on FISH variables.

Studied groups	The percentage of correct classification	Healthy controls	Disease phases	
			Remission	Attack
Healthy controls	100.0% (35.0%)*	7	0	0
FMF remission	83.3% (30.0%)	0	5	1
FMF attack	100.0% (35.0%)	0	0	7
Total	95.0% (33.3%)	7	5	8

Statistical parameters of the discriminant model: Wilks's lambda coefficient – 0.045. Significance of the model: F (20,16) = 2.95; p<0.016.

* *apriori* probability of correct classification.

## Discussion

This is the first work reporting the specific changes in the human gut microbiota due to the mutations in a single gene of the host. Suggestions that the composition of gut microbiota is host genotype-dependant have been proposed earlier [43], [44], however, these works were based on twin studies and the host genotype was not assessed. Gut inflammatory diseases such as IBD have been long known as having a substantial genetic predisposition component and a recent review of genome-wide scans reported on the seven confirmed and replicated cases of genetic susceptibility loci, with many more, however, that need independent confirmation and verification [42]. Although the gut microbiota of IBD patients was subjected to several diversity analyses [35], [36], [45], the host genetics was not assessed, most probably because of complicated genetics of IBD. Thus, uncovering the genetic mechanisms that are involved in regulation of composition of human gut microbiota remains a challenging task. Evidence obtained in a study of bacterium-derived cellular fatty acids in the stool samples of six mouse strains congenic for the major histocompatibility complex (MHC) suggested the importance of host immune system in this process [46]. The obvious advantage of our model is that the genetics of FMF is well defined and confined to a single gene thus facilitating the host genotype analysis. Thus, for the first time, we had the opportunity for direct comparison of the host and gut community genotypes.

Initially, we genotyped the FMF patients and healthy controls for the presence of disease-specific mutations in exons 2 and 10 of the *MEFV* gene. In all clinically confirmed cases we were able to detect the presence of known mutations, all of them in exon 10, which encodes the SPRY/B30.2 domain. Among our very limited cohort of seven healthy controls of Armenian origin, one heterozygote was detected, suggesting that the mutation carrier rate in this population is at least 14%, which is within the range of carrier rates found in populations of other ethnic origin [47]–[50]. The high carrier frequency in affected populations suggests the selective advantage conferred by the heterozygous state but no perceptible biological advantage of the carrier state is presently identified [49]. Positive selection to maintain the high frequency of the heterozygotes should also overcome the negative selection imposed by the increased morbidity and mortality rates among the homozygotes and compounded heterozygotes. Clinical manifestations during the disease attack are consistent with a strong inflammatory response, with fever, massive influx of polymorphonuclear leukocytes into the affected tissues, neutrophilia, and acute-phase response. In some patients this leads to systemic amyloidosis resulting in renal failure and death.

The penetrance of mutations that are all missense mutations, varied widely. Apart from the combined heterozygotes, which support the recessive nature of mutations, there were also simple heterozygotes but with different phenotypes. The simple heterozygosity in clinically confirmed disease was exclusively exemplified by a M694V mutation and, similarly to another study [51], suggested the autosomal dominant inheritance probably due to the importance of this residue in the interaction of pyrin with caspase-1 [52]. On the other end of the mutation spectrum is the simple M680I heterozygosity found in one of the healthy volunteers, C6b, which did not result in any clinical or subclinical (cytokine profile) FMF manifestation. Thus, depending on the locus involved, the outcome could be either an asymptomatic carrier state or a full-blown disease. In the case of M694V mutation, a 50% complementation by the normal allele may be not sufficient to provide the protection against clinical manifestations of the disease. This is the most abundant mutation in our FMF cohort and is present in 70% of the patients. Taking into the consideration the high penetrance and associated fitness cost of carrying this allele even in the heterozygous form it seems counterintuitive to suggest that it may confer any selective advantage in the heterozygous carrier state under the present day living conditions. Phenotypically, the asymptomatic heterozygous carriers of FMF mutations are characterized by the elevated levels of markers of inflammation such as CRP, SAA, and INF-γ [53], [54] and by specific glycosylation of AGP [55]. Whether this subclinical inflammation in asymptomatic patients may be protective against specific pathogens, probably endemic for the Mediterranean basin, remains to be seen since the previous studies failed to identify such agents [49].

In a parallel study, we measured the levels of systemic markers of inflammation in acute phase and remission of FMF [56]. The study confirmed earlier observations that even in remission, where the clinical signs of disease are absent, the level of certain markers of inflammation remains elevated suggesting chronic subclinical inflammation in FMF. How this inflammation may affect the gut microbiota? To answer this question, we used two independent techniques to assess gut molecular bacterial diversity in health and disease, the sequence analysis of 16S rRNA gene libraries and FISH. Both these techniques have their own advantages and disadvantages but in combination they are complementary and allow obtaining a more balanced overview of gut microbiota. In particular, 16S libraries may suffer from PCR biases introduced by elevated number of cycles [57] or poor amplification of certain templates [58] while FISH analysis is less sensitive since it targets higher taxonomic entities and no community analysis involving individuals and species/phylotypes, unless they very are numerous, is possible. On the other hand, FISH technique consistently detects higher levels of *Bifidobacteria* than clone library sequencing, it is less expensive and can be easily automated in conjunction with flow cytometry or computer-aided automatic count under the microscope for analysis of large cohorts. In order to minimize inter-individual variations, 16S rRNA gene libraries were constructed from fecal samples of 12 FMF patients in remission, three patients in the acute stage of FMF, six healthy Armenians, and one healthy subject of non-Armenian origin. The number of amplification cycles was maintained low (10 cycles) to prevent a potential PCR bias.

Both analyses of gut microbiota produced the similar results in terms of general distribution of major phyla representatives in the human gut. Similarly to the previous studies [8], [36], [59] the vast majority of bacterial sequences and cells hybridized to specific probes in the gut fell into the *Bacteroidetes* and *Firmicutes* phyla. The *Bacteroidetes* contained the largest number of sequences but fewer phylotypes while the diverse *Firmicutes* were dominant in terms of the numbers and the relative proportion of phylotypes detected. The *Bacteroidetes* was also the largest group detected by the Bac303 probe accounting for 27–39% of the total bacterial count, which is lower than the clone library estimates. On the contrary, the combination of various FISH probes targeting the *Firmicutes* produced a higher estimate of bacteria belonging to this phylum than the clone libraries. The most profound difference between the two techniques in detection of bacteria in the human gut, however, concerned the enumeration of high G+C Gram-positive bacteria such as *Atopobium* spp. and *Bifidobacterium* spp.: FISH technique consistently detected a sizable population of these genera in the gut while they were hardly represented in PCR-generated libraries. Taking into the consideration an important role played by, for example, *Bifidobacteria* in maintaining the gastrointestinal health, consistent underestimates of these bacteria in clone library analyses may obscure the effect of this group in various gut diseases such as IBD as well as in the healthy gut.

Examination of gut bacterial diversity in healthy and diseased cohorts by the two methods arrived essentially at the same conclusion suggesting the specific shifts in composition of bacteria imposed by mutations in the *MEFV* gene of the host. The gut microbiota in active disease was characterized by diminished bacterial diversity and major population restructuring within the phyla. In particular, the proportion of phylotypes belonging to of *Porphyromonadaceae*, *Phascolarctobacterium*, *Faecalibacterium*, and *Parabacteroides* were significantly increased while *Prevotellaceae, Dialister* and *Prevotella* were significantly lower in acute disease in comparison with control. Both approaches detected the fall in proportion of clostridial cluster IX in FMF attack: FISH demonstrated it for the whole group, while the sequence libraries elaborated this general trend as involving *Acidaminococcaceae, Megasphaera,* and *Dialister.* Evenness values for microbiota in active disease were higher reflecting the lower number of dominant phylotypes, although this could be a consequence of the smaller number of sequences collected. Also, in a parallel FISH approach, the fall in absolute numbers of bacteria during the inflammation attacks was detected. In most cases, this analysis is less sensitive than sequencing because it targets the larger taxonomic entities such as, for example, the whole *Bacteroides* group and cannot detect the differences at the lower taxa levels, e.g., in the *Prevotellaceae* and *Porphyromonadaceae* families and the *Prevotella* and *Parabacteroides* genera while sequence analysis operating at the individual sequence level is capable to do. In some cases, however, the situation may be opposite: Rfla729/Rbro730 FISH probe, for example, detects a subset of ruminococci, which is significantly reduced in attack (Table 5), while at the genus level the differences are not significant (Table 3). Thus, FISH served as a complementary tool and allowed analyzing bacterial populations that are routinely underestimated in sequence analyses. In particular, FISH detected the fall in absolute numbers of high G+C bacteria such as *Bifidobacterium* and *Atopobium*, which are not represented well in our and others SSU rRNA clone libraries [59]. Thus, in disease attack, the drop of total bacterial count and restructuring of bacterial composition in the gut may be the consequence of acute inflammation, which is accompanied by fever and influx of polymorphonuclear neutrophils (PMNs) into the affected areas including peritoneum. Elevated body temperatures (up to 40°C) and the localized respiratory burst of PMNs producing reactive oxygen species could be responsible for gut microbiota selection.

As mentioned earlier, despite the absence of any clinical signs of disease in remission, certain systemic markers of inflammation are still elevated suggesting subclinical chronic inflammation [56]. Interestingly, the diversity values in remission were higher than in the healthy gut suggesting that the low-level subclinical inflammation in FMF may favour a greater diversity of intestinal bacteria. DA analysis of FISH data also confirmed the specific composition of bacterial populations in remission, which is different from control and forms a separate cluster. An additional internal control subject of different ethnic background was included in our study and the classification of the subject was consistently in the healthy group. The specific shifts of bacterial composition were also confirmed in two patients that were investigated during the remission and attack periods to exclude the influence of other genetic or environmental factors and these analyses confirmed the specific bacterial composition shifts due to disease state such as the increase of butyrate producers and the fall of clostridial cluster IX bacteria during disease attacks. While these shifts in attack may be explained by factors such as acute inflammation, with fever and PMN infiltration, the factors that govern the specific bacterial shifts in remission, which is free of disease symptoms, are harder to suggest. Nevertheless, our DA model consistently classified the patients in remission as a separate group, which suggests the specific patterns of gut microbiota in this cohort.

Thus the question is: what are the molecular mechanisms responsible for these microbiota shifts imposed by the mutated version of pyrin? In a broad sense, the functional role of this protein is the regulation of innate immunity [38]. The protein is expressed in granulocytes, activated monocytes, and serosal and synovial fibroblasts [60]–[62]. Recently it becomes evident that the regulatory functions of pyrin are carried out through the interaction of two domains of the protein, PYRIN and B30.2/rfp/SPRY, with the proinflammatory caspase-1 activating complex, called the inflammasome. The N-terminal PYRIN domain of the protein binds and competes for ASC (apoptosis-associated speck-like protein containing a caspase-recruitment domain), an inflammasome component, thereby decreasing its availability for the cryopyrin/NALP3/CIAS1 inflammasome. This results in reduction of caspase-1 activation and pro-interleukin-1beta processing and secretion thus halting the inflammatory response [63]. The mutational hotspot in FMF, however, is located in the C-terminal B30.2/rfp/SPRY domain, which modulates the inflammatory response through the interaction with several components of the inflammasome. In addition to interaction with cryopyrin/NALP3/CIAS1, it interacts directly with caspase-1 and its substrate pro-IL-1β [52], [64]. The net effect of these interactions is the suppression of IL-1β activation and block of inflammation. It was suggested that the heightened IL-1β responsiveness could be a factor selecting for mutations in the MEFV gene [52]. Taking into consideration the overall role of pyrin as a modulator/suppressor of the inflammatory response, another factor contributing to the autoinflammatory nature of the disease could be the reduced *MEFV* messenger RNA expression in patients with familial Mediterranean fever [65]. First, it may contribute to the lower titre of pyrin and its PYRIN domain molecules in the cell thus making more ASC molecules available to initiate caspase-1 activation. Second, the reduced concentration of pyrin and therefore of its B30.2/rfp/SPRY domain, which, in addition, is mutated to the loss of caspase-1 suppressor function in most of FMF cases, may provoke easier triggering the inflammation cascade through caspase-1 activation. Thus, both consequences of *MEFV* mutations may lead to the heightened responsiveness of cryopyrin/NALP3/CIAS1, which can be oligomerized and activated in response to a very diverse range of ligands such as bacterial muramyl dipeptide, ATP, toxins, bacterial and viral RNA, small antiviral compounds, *Staphylococcus, Listeria*, and uric acid crystals as well as by low intracellualr potassium concentration [66]–[71]. While these exo- and endogenous stimuli are normally not perceived as danger signals or are efficiently suppressed, the facilitated oligomerization of the cryopyrin/NALP3/CIAS1, which is not adequately suppressed by the mutated pyrin in FMF, may be the underlying cause in this disease, resulting in excessive pro-caspase-1 and pro-IL1β processing. Indeed, monocytes from FMF patients in remission fail to induce LPS homologous tolerance and exhibit heightened sensitivity to bacterial endotoxin [72], one of the important activators of the cryopyrin/NALP3/CIAS1 inflammasome [73].

In conclusion, we established in this work that mutations in a single host gene lead to specific restructuring of commensal gut microbiota. Although the exact molecular mechanisms behind this process are not fully understood, at our current genetic stage of analysis the work points to the host genotype as a key factor in symbiotic host-microbe interaction. Further developments in this area may focus on details of this interaction at the biochemical and physiological levels. Other models of gut inflammation such as IBD may help to establish the role of other host genes affecting the composition of commensal gut microbiota. Comparative community analyses from genetically different hosts are particularly interesting in identifying gut microbiota alterations specific for a particular disease genotype. At the same time, it cannot be excluded that the changes in the community structure may share some common features reflecting the similarities in disease phenotypes such as inflammation. And finally, how the gut microbiota is transformed in disease to become a “pathobiota” [37] remains an interesting question to answer.

## Materials and Methods

### Subjects and sampling

A total of 19 Armenian FMF patients with clinically confirmed disease, seven healthy individuals of the same ethnic background, and one healthy subject of non-Armenian origin participated in this study (Table 1). All FMF cases were diagnosed based on Tel-Hashomer criteria [74]. Blood and fecal samples were collected from FMF patients (15 males, four females; aged from 15 to 64 years, mean age–27 years) and control subjects (one male, seven females; aged from 32 to 67 years, mean age–43.8 years) for genotyping and gut bacterial diversity analyses (see below). None of the FMF patients and healthy individuals had used antibiotics within the three months prior to sampling. In the beginning of this study, ten FMF patients were not receiving any drugs and nine were undergoing regular colchicine therapy. During the study, one patient from the colchicine-free group was prescribed this therapy. All subjects were informed about the aim of this study and gave their consent to participate in it. The study was approved by the local ethical committee at IMB.

### Genetic diagnostics

Venous blood samples were obtained from the clinically confirmed 19 FMF patients and eight healthy controls to reveal the *MEFV* mutation carrier status. Genetic analysis was based on screening of two mutational hot spots in the *MEFV* gene (exons 2 and 10). For this, genomic DNA was isolated from the anticoagulated venous blood samples using the Wizard Genomics DNA Purification kit (Promega, UK), according to the manufacturer's instructions. Exon 2 of the *MEFV* gene was PCR amplified from genomic DNA using a newly designed forward and reverse primer set: Exon2F - 5′-ATTCTCTCTCCTCTGCCCTG-3′, Exon2R - 5′-CCATTCTTTCTCTGCAGCCG-3′, yielding a 839 bp amplicon. Exon 10 was amplified using the primers Exon10F 5′-CCAGAAGAACTACCCTGTCCC-3′ and Exon10R 5′-TCCTCCTCTGAAATCCATGG-3′, yielding a product of 887 bp [39]. For exon 10, amplification was performed in a final volume of 50 µl, in a mix containing 10 mM Tris-HCl, 1.5 mM MgCl_2_, 50 mM KCl, 200 µM of each dNTP, 10.0 pmol of each primer, approximately 20 ng of genomic DNA, and 1 U of Red Taq polymerase (Bioline, UK). The cycling conditions included the initial denaturation step for 5 min at 94°C, followed by 35 cycles consisting of denaturation at 94°C for 30 sec, annealing at 61°C for 60 sec, and extension at 72°C for 60 sec, with a final extension at 72°C for 10 min. For exon 2, PCR amplification was performed similarly, except that the PCR mix contained 1 M betaine (Sigma-Aldrich, UK). Amplicons of the expected size were excised from 1.5% agarose gel and purified using a Wizard SV Gel and PCR Clean-up system (Promega, UK), according to the manufacturer's instructions. Purified PCR products were sequenced on both directions, using the same primer set as for generation of templates and the sequences were read on an automated 8-channel capillary sequencer (Beckman, USA) The wild-type sequences of exons 2 and 10 of the *MEFV* gene were retrieved from GenBank (accession number AF111163).

### Generation of 16S rDNA clone libraries

In order to derive a detailed phylogenetic inventory of the gut microflora in FMF and in healthy controls, we constructed bacterial 16S rDNA gene libraries from fecal samples. For this, freshly voided feces were collected into sterile containers from 12 FMF patients in remission, three patients in the acute stage of FMF, six healthy Armenians, and one healthy subject of non-Armenian origin (Table 1). One patient, FMF 13-123, provided the samples both in remission and attack. Total DNA was extracted from fecal samples using QIAamp DNA Stool Mini Kit (Qiagen, UK), according to the manufacturer's instructions. DNA was additionally purified by phenol-chloroform-isoamyl alcohol (25∶24∶1) extraction, ethanol precipitated, and dissolved in TE buffer. Amplification of the 16S rRNA genes was carried out with the universal primer set–27F (5′-AGAGTTTGATCMTGGCTCAG-3′; positions 8 to 27 in the *Escherichia coli* 16S rRNA gene) and 1492R (5′-ACGGCTACCTTGTTACGACTT-3′; positions 1510 to 1492 in the *E. coli* 16S rRNA gene) [75]. The PCR reaction mixture contained 10 mM Tris-HCl, 2 mM MgCl_2_, 50 mM KCl, 200 µM of each dNTP, 10.0 pmol of each primer, 1U of Bio-X-Act Short DNA polymerase (Bioline, UK), and 100 ng of template DNA in a final volume of 50 µl. To reduce PCR bias caused by elevated number of PCR cycles [57], amplifications were performed using the following conditions: initial denaturation of template DNA at 94°C for 5 min, followed by 10 cycles of denaturation at 94°C for 30 sec, annealing at 57°C for 30 sec, and extension at 72°C for 2 min, with a final extension at 72°C for 10 min. PCR products were visualized on 1% agarose gel in TBE buffer stained with GelStar (Cambrex, UK). 16S PCR products were purified using Wizard SV Gel and PCR Clean-up system (Promega, UK), according to the manufacturer's instructions. This was followed by ethanol precipitation and the final pellets were suspended in 5 µl of TE buffer (pH 8.0). The purified PCR products were directly cloned into pCR-4 cloning vector and then transformed into *E. coli* TOP10 chemically competent cells using the TOPO TA Cloning Kit (Invitrogen, UK), according to the manufacturer's protocols. Colonies were blue/white screened on Luria-Bertani (LB) agar plates supplemented with ampicillin (50 µg/ml) and X-Gal (80 µg/ml). No IPTG was necessary in this system because the strain and plasmid do not carry the *lac* repressor. White recombinant colonies were randomly picked up into 200 µl of LB medium with ampicillin (50 µg/ml) in a 96-well format, sealed with a gas-permeable membrane, and incubated overnight at 37°C in a horizontal shaker. Two insert-surrounding primers, T3 (5′-ATTAACCCTCACTAAAGGGA-3′) and T7 (5′-TAATACGACTCACTATAGGG-3′) were used to amplify inserts using a 35-cycle colony PCR. Products were visualized after electrophoresis on 1% agarose gel in TAE buffer and ethidium bromide staining.

### Sequencing and phylogenetic analysis

Successfully amplified 16S rDNA inserts were purified and sequenced on the automated 8-channel capillary sequencers (Beckman, USA). The sequencing reactions were carried out with 926R primer (5′-CCGTCAATTCCTTTGAGTTT-3′; positions 926 to 907 in the *E. coli* 16S rRNA gene) [76]. Nucleotide sequences were aligned using the multiple sequence alignment program CLUSTALX v1.83 [77]. Each sequence was manually edited in conjunction with its chromatogram. Sequences were examined for the possible chimeras using CHIMERA_CHECK online analysis at RDP-II website (http://rdp.cme.msu.edu/cgis/chimera.cgisuSSU). Similarity search of the 16S rDNA sequences against database entries was performed using online Basic Local Alignment Search Tool (BLAST) program at the NCBI website (http://www.ncbi.nlm.nih.gov/BLAST). Distance and similarity matrices were constructed with the DNADIST program v3.6 from PHYLIP (http://evolution.genetics.washington.edu/phylip/phylip.html) according to the Jukes-Cantor model. Phylogenetic analysis was performed using the neighbour-joining method [78]. Statistical significance of branching was verified by bootstrap analysis involving the construction and analysis of 1000 trees from bootstrapped data sets. Operational taxonomic units (OTUs), or phylotypes, were defined at 99% level as recommended for gut microbiota analyses [59]. To assign sequences quickly and accurately to OTUs, additionally, Jukes-Cantor corrected distance matrices were analyzed with DOTUR (Distance-Based OTU and Richness) by using the furthest-neighbor method [79].

About the half of OTUs (165 out of 346) were sequenced to completion. For this, a set of additional three universal bacterial primers, 27F (5′-AGAGTTTGATCMTGGCTCAG-3′; positions 8 to 27 in the *E. coli* 16S rRNA gene), 519R (5′-GTATTACCGCGGCTGCTG-3′; positions 536 to 519 in the *E. coli* 16S rRNA gene) [76] and RP2 (5′- ACGGCTACCTTGTTACGACTT -3′; positions 1510 to 1492 in the *E. coli* 16S rRNA gene) was used. These partial rRNA gene sequences (the average single sequence read length was ∼700 bp), including the sequence previously generated by the 926R primer, were assembled with ChromasPro v1.33 program to produce a consensus sequence for each of the 165 clones. Initially, all sequence data were assembled according to their overlapping sequence fragments into a contiguous consensus sequence using default program settings or, in some cases, adjusting the program settings, if necessary, followed by verification by eye. Afterwards, all chromatogram contigs obtained were manually edited removing ambiguous chromatogram areas, which resulted in the nearly full-length 16S bacterial rDNA sequences of about 1500 bp.

All full-length sequences were checked for possible chimeras using Chimera Check program, v2.7 (online analysis at Ribosomal Database Project II website, http://rdp.cme.msu.edu/cgis/chimera.cgisuSSU). Chimeras and sequences of poor quality were omitted from further phylogenetic analyses. Phylogenetic analysis was performed in two stages. First, the almost full-length sequences from our libraries were combined with the closest relatives, cultivated and uncultivated, from databases, aligned, and subjected to phylogenetic analysis using the neighbor-joining method [78]. In the second stage, the partial sequences were aligned to the full-length sequence profile using the guide tree generated during the first stage. A total of 1328 validated 16S rDNA sequences participated in the final tree computing. Validation of the tree branching was done with bootstrap analysis [80] involving 1,000 reiterations with re-sampled data sets.

### Community structure analysis

Rarefaction and collector's curves of observed phylotypes, richness estimates and diversity indices were determined with DOTUR program using Jukes-Cantor corrected distance matrix. The bias-corrected Chao 1 richness estimator was calculated after 1000 randomizations of sampling without replacement. Collector's curves of observed and estimated (Chao 1 and the abundance-based coverage estimator, ACE) richness were constructed. Diversity was estimated by the Shannon and Simpson indices, the Simpson reciprocal index was calculated as 1/D, and another version of the Simpson diversity index–as 1-D. The evenness was calculated as E = e^H^/N, where H is the Shannon diversity index, N is the number of phylotypes. The Good's coverage percentage was calculated with the formula [1−(n/N)]×100, where n is the number of phylotypes in a sample represented by one clone (singletons) and N is the total number of sequences in that sample [81].

### Fluorescent in situ hybridization (FISH)

FISH analysis was applied to quantify the predominant groups of bacteria in fecal samples using the universal bacterial and group-specific 16S rRNA-based Cy3-labelled oligonucleotide probes (Table 9). The probes hybridized with the next bacterial groups: Eub338 with the total bacteria [82]; Bac303 with the *Bacteroides* group [83]; Erec482 with the members of the *Clostridium coccoides* group (cluster XIVa) [84]; Rrec584 with the *Eubacterium rectale-Roseburia* cluster, which is a component of clostridial cluster XIVa [85]; Fprau645 for *Faecalibacterium prausnitzii*-related bacteria–a component of clostridial cluster IV [86]; the Rfla729 and the Rbro730 probes are specific for ruminococci in clostridial cluster IV [87]; Prop853 for the members of clostridial cluster IX [88]; Bif164 for the genus *Bifidobacterium* [89]; Ato291 for the *Atopobium* group [90]; EnterobactD for the genus *Enterobacteriaceae* [91]; and Lab158 for lactobacilli and enterococci [92] (Table 9). Together, the group-specific probes detected the vast majority of the total bacterial cells in the normal human gut [84]. The Rrec584 probe was excluded from this summation, since it detects the *E. rectale-Roseburia* cluster within the group already covered by the probe Erec482 [85]. The probe Rfla729 was used in conjunction with the probe Rbro730 as the target bacterial groups are overlapping [87].

**Table 9:** Oligonucleotide probes and hybridization conditions used in this study.

Probe	Sequence (5′-3′)	Targeted bacteria	Hybridization conditions*		
			T	F	L
Eub338	GCTGCCTCCCGTAGGAGT	*Bacteria*	50	-	-
Bac303	CCAATGTGGGGGACCTT	*Bacteroides* group	47	-	-
Erec482	GCTTCTTAGTCAGGTACCG	Clostridial cluster XIVa	47	-	-
Rrec584	TCAGACTTGCCG(C/T)ACCGC	*Roseburia-E. rectale* group	50	-	-
Fprau645	CCTCTGCACTACTCAAGAAAAAC	*F. prausnitzii* group	50	-	-
Rfla729	AAAGCCCAGTAAGCCGCC	*R. flavefaciens* subcluster	50	20	10
Rbro730	TAAAGCCCAG(C/T)AGGCCGC	*R. bromii* sublcuster	50	20	10
Prop853	ATTGCGTTAACTCCGGCAC	Clostridial cluster IX	50	-	-
Bif164	CATCCGGCATTACCACCC	*Bifidobacterium* genus	50	-	-
Ato291	GGTCGGTCTCTCAACCC	*Atopobium* group	50	-	-
EnterobactD	TGCTCTCGCGAGGTCGCTTCTCTT	*Enterobacteriaceae* genus	50	-	-
Lab158	GGTATTAGCA(C/T)CTGTTTCCA	*Lactobacillus-Enterococcus*	45	20	10

* T, Hybridization temperature (°C); F, Formamide concentration (%); L, Lysozyme treatment (min).

Freshly voided feces from six FMF patients in remission, seven in the acute phase, six healthy Armenians and one healthy subject of non-Armenian descent were collected (Table 1). Two patients, FMF 13-123 and FMF 87, provided fecal samples at both disease stages. The samples in sterile plastic bags were kept at 4°C for no longer than 12 h prior processing. The specimens were kneaded mechanically for 5 min at 4°C to distribute the sample evenly. 0.5 g of each sample was suspended in 4.5 ml of filtered (0.2 µm pore-size filter) ice-cold phosphate-buffer-saline (PBS) and vortexed with a dozen of glass beads for at least 3 min to homogenize the sample and dislodge the bacteria from feed particles. The suspension was centrifuged at 700×g for 1 min to remove debris. One ml of supernatant was added to three ml of 4% paraformaldehyde (PFA) in PBS, fixed at 4°C for 16 hours, washed with ice-cold PBS, and stored in 50% (vol/vol) ethanol-PBS at −20°C until analysed.

Depending on the expected number of target cells, the samples were diluted 40- to 1600-fold, and 10 µl of a diluted bacterial suspension was applied to gelatin-coated slides, air-dried and fixed in 100% ethanol. 10 µl of 50 ng/µl solution of oligonucleotide probes in 100 µl of hybridization buffer was added to the slides and the samples were hybridized overnight (except for the Bac303 probe, which was incubated for 2 h) in a precision incubator at different temperatures, depending on the probe (Table 9). If more stringent conditions were required, formamide was added to the hybridization buffer. To improve bacterial cell permeability samples were incubated before hybridization in 10 µl of lysozyme (final concentration 1 mg/ml) in 100 mM Tris-HCl (pH 8.5) at 37°C. The full panel of FISH oligonucleotide probes with their target bacterial groups and hybridization conditions used in this study is shown in Table 9.

After hybridization, the slides were soaked in a washing buffer for 20 min at 50°C, rinsed with Milli-Q water, and rapidly dried with compressed air. Slides then were covered with 50 µl of Vectashield (Vector Laboratories, Burlington, California) to prevent the fading of fluorescence and a coverslip. The enumeration was performed when the hybridization signals were clear and morphologically distinguishable. Bacterial cells were counted automatically using image analysis software Quantimet HR600 with a Leica DMRXA epifluorescence microscope (Wetzlar, Germany). Depending on the number of labelled cells, from 20 to 50 fields were counted to bring the coefficient of variance value below 10.

### Statistical analyses

Statistical analyses were performed using the Statsoft Statistica package (www.statsoft.com). Comparisons were made among groups of subjects using the Mann-Whitney *U* test and Student's t-test. Multiple comparisons were carried out using Kruskal-Wallis test. In order to classify cases data were subjected to discriminant function analysis (DA). The P-values below 0.05 were considered statistically significant.

Statistical comparison of 16S rDNA clone libraries was performed using online library compare tool at RDP-II website (http://rdp.cme.msu.edu/comparison/comp.jsp). This tool uses the RDP Naive Bayesian classifier to provide rapid classification of library sequences into the bacterial taxonomy proposed by the Bergey's Trust. The P values <0.01 were considered as having significant differences.

**Data deposition:** The sequences reported in this paper have been deposited in GenBank with accession numbers EU531867-EU532010 and EU532765-EU533948.
